# The McDonald exponentiated gamma distribution and its statistical properties

**DOI:** 10.1186/2193-1801-4-2

**Published:** 2015-02-12

**Authors:** Abdulhakim A Al-Babtain, Faton Merovci, Ibrahim Elbatal

**Affiliations:** 1 Statistics and Operations Research, College of Science, King Saud University, P.O. Box 2455, Riyadh, 11451 Saudi Arabia; 2 Department of Mathematics, University of Prishtina “Hasan Prishtina” & University of Mitrovica “Isa Boletini”, Mother Teresa, Av=5, 10000 Prishtinë, Kosovo; 3 Institute of Statistical Studies and Research, Department of Mathematical Statistics, Cairo University, Cairo, Egypt

**Keywords:** McDonald exponentiated gamma distribution, Moments, Exponentiated gamma distribution, Order statistics, Maximum likelihood estimation

## Abstract

**Abstract:**

In this paper, we propose a five-parameter lifetime model called the McDonald exponentiated gamma distribution to extend beta exponentiated gamma, Kumaraswamy exponentiated gamma and exponentiated gamma, among several other models. We provide a comprehensive mathematical treatment of this distribution. We derive the moment generating function and the rth moment. We discuss estimation of the parameters by maximum likelihood and provide the information matrix.

**AMS Subject Classification:**

Primary 62N05; secondary 90B25

## 1 Introduction

The gamma distribution is the most popular model for analyzing skewed data and hydrological processes. One of the important families of distributions in lifetime tests is the exponentiated gamma (EG) distribution. The exponentiated gamma (EG) distribution has been introduced by Gupta et al. 1998 which has cumulative distribution function (c.d.f.) and a probability density function (p.d.f.) of the form, respectively;
1


where *λ* and *θ* are scale and shape parameters respectively. The corresponding probability density function (pdf) is given by
2


Shawky and Bakoban 2008 discussed the exponentiated gamma distribution as an important model of life time models and derived Bayesian and non-Bayesian estimators of the shape parameter, reliability and failure rate functions in the case of complete and type-II censored samples. Also order statistics from exponentiated gamma distribution and associated inference was discussed by Shawky and Bakoban 2009. Ghanizadeh, et al. 2011, dealt with the estimation of parameters of the exponentiated gamma (EG) distribution with presence of *k* outliers. The maximum likelihood and moment estimators were derived. These estimators are compared empirically using Monte Carlo simulation. Singh et al. 2011b proposed Bayes estimators of the parameter of the exponentiated gamma distribution and associated reliability function under general entropy loss function for a censored sample. The proposed estimators were compared with the corresponding Bayes estimators obtained under squared error loss function and maximum likelihood estimators through their simulated risks. Khan and Kumar 2011established the explicit expressions and some recurrence relations for single and product moments of lower generalized order statistics from exponentiated gamma distribution. Sing et al. 2011a where proposed Bayes estimators of the parameter of the exponentiated gamma distribution and associated reliability function under general entropy loss function for a censored sample. Feroze ans Aslam 2012 introduced Bayesian analysis of exponentiated gamma distribution under type II censored samples. Recently, Nasiri et al. 2013 discussed Classical and Bayesian estimation of parameters on the generalized exponentiated gamma distribution.

## 2 Mc-Donald generalized distribution

Consider an arbitrary parent cdf *G*(*x*). The probability density function (pdf) *f*(*x*) of the new class of distributions called the Mc-Donald generalized distributions (denoted with the prefix "Mc" for short) is defined by
3


where *a* > 0,*b* > 0 and *c* > 0 are additional shape parameters. (See Corderio et al. (2012) for additional details). Note that *g*(*x*) is the pdf of parent distribution, . Introduction of this additional shape parameters is specially to introduce skewness. Also, this allows us to vary tail weight. It is important to note that for *c* = 1 we obtain a sub-model of this generalization which is a beta generalization (see Eugene et al. 2002) and for *a* = 1, we have the Kumaraswamy (Kw), [Kumaraswamy generalized distributions (see Cordeiro and Castro 2011)). For random variable *X* with density function (2), we write *X* ∼ *M* *c*-*G*. The probability density function (3) will be most tractable when *G*(*x*) and *g*(*x*) have simple analytic expressions. The corresponding cumulative function for this generalization is given by
4


where  denotes the incomplete beta function ratio (Gradshteyn and Ryzhik 2000). Equation (4) can also be rewritten as follows
5


where



is the well-known hypergeometric functions which are well established in the literature (see, Gradshteyn and Ryzhik 2000). Some mathematical properties of the cdf *F*(*x*) for any Mc-G distribution defined from a parent *G*(*x*) in Equation 5, could, in principle, follow from the properties of the hypergeometric function, which are well established in the literature (Gradshteyn and Ryzhik 2000 Sec. 9.1). One important benefit of this class is its ability to skewed data that cannot properly be fitted by many other existing distributions. Mc- G family of densities allows for higher levels of flexibility of its tails and has a lot of applications in various fields including economics, finance, reliability, engineering, biology and medicine.

The hazard function (hf) and reverse hazard functions (rhf) of the Mc-G distribution are given by
6


and



respectively. Recently Cordeiro et al. 2012 presented results on the McDonald normal distribution. Cordeiro et al. 2012 proposed McDonald Weibull distribution, Merovci and Elbatal 2013 proposed McDonald modified Weibull distribution, Elbatal et al. 2014 proposed McDonald generalized linear failure rate Distribution, Elbatal and Merovci 2014 introduced McDonald Pareto distribution and Marciano et al. 2012 obtained the statistical properties of the *Mc* - Γ and applied the model to reliability data. In this paper we introduce a new class of distribution, called McDonald exponentiated gamma (*McEG*) distribution which extends the exponentiated gamma model and has several other models as special cases. since it has more shape parameters, yielding a large variety of forms. It can also be useful for testing the goodness of fit of its sub-models.

The outline of this paper is as follows. In Section 2, the McDonald exponentiated gamma (*MceG*) and related family distributions are introduced. The series expansion for the density, hazard and reverse hazard functions, and other properties are presented in Section 3. Section 4 provides expansions for the cumulative and density functions. In Section 5, we present the statistical properties, in particular moments, moment generating function. The distribution of the order statistics is expressed in Section 6. Section 7 provides least squares and weighted least squares estimators. Maximum likelihood estimates of the parameters index to the distribution are discussed in Section 8. Section 9 provides applications to real data sets. Section 10 ends with some conclusions.

## 3 McDonald exponentiated gamma distribution

In this section we studied the five parameter McDonald exponentiated gamma (*McEG*) distribution. Using *G*(*x*) and *g*(*x*) in (3) to be the cdf and pdf of (1) and (2). The pdf of the *McEG* distribution is given by
7


where *x* > 0 and *φ* = (*λ*,*θ*,*a*,*b*,*c*). The corresponding cdf of the *McEG* distribution is given by
8


also, the cdf can be written as follows
9


where 

**Figure 1:**
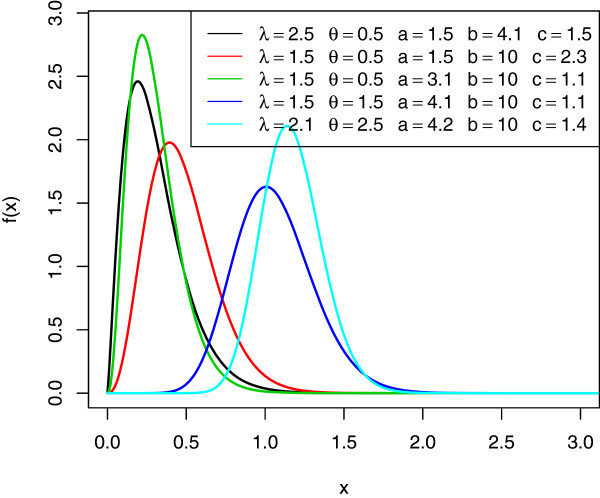
**The pdf’s of various McEG distributions.**

**Figure 2:**
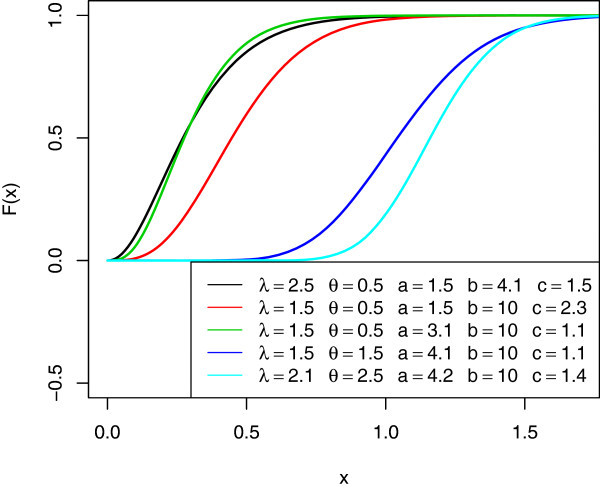
**The cdf’s of various McEG distributions.**

Figures 1 and 2 illustrates some of the possible shapes of the pdf and cdf of the McEG distribution for selected values of the parameters *λ*,*θ*,*a*,*b* and *c*, respectively.

The hazard rate function and reversed hazard rate function of the new distribution are given by
10


and
11


respectively.

**Figure 3:**
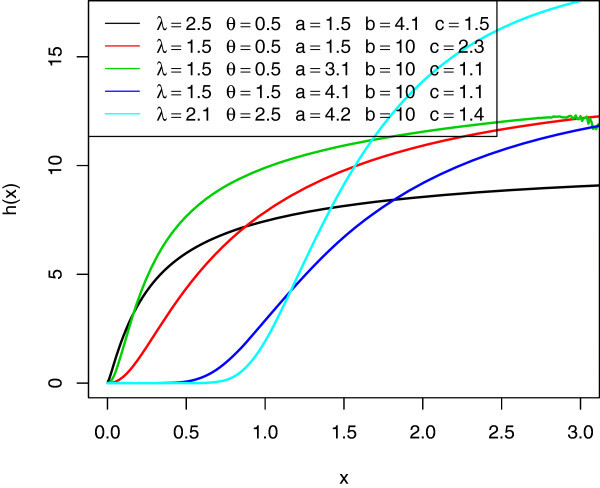
**The hazard rate’s of various McEG distributions.**

**Figure 4:**
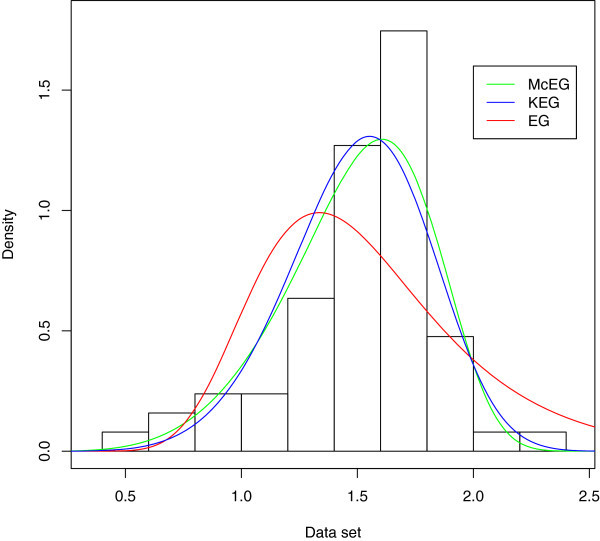
**The reversed hazard rate’s of various McEG distributions.**

Figures 3 and 4 illustrates some of the possible shapes of the hazard and reversed hazard of the McEG distribution for selected values of the parameters *λ*,*θ*,*a*,*b* and *c*, respectively.

## 4 Expansions for the cumulative and density functions

In this section,we present a series expansion of the *McEG* cdf and pdf. distribution depending if the parameter *b* > 0 is real non- integer or integer. First, if |*z*| < 1 and *b* > 0 is real non- integer, we have in this subsection, we present some representations of cdf and pdf of (*McEG*) Equations 7 and (8) are straightforward to compute using any software with algebraic facilities. The mathematical relation given below will be useful in this subsection. If *b* is a positive real non integer and |*z*| ≤ 1,then
12


Using the expansion (12) in (8), the cdf of the *McEG* distribution becomes
13


If *b* > 0 is an integer, then
14


Similarly, if *b* > 0 is real non- integer the pdf is given by



and
15


for *b* > 0 is an integer. Where  are constants such that  and *G*(*x*,*λ*,*θ* *c*(*a* + *j*)) is a finite mixture of exponentiated gamma distribution with *λ* and *θ* *c*(*a* + *j*) are scale and shape parameters respectively. The graphs below are the pdf, cdf, survival function, *h*(*x*), and *τ*(*x*) of the *McEG* distribution for different values of parameters *λ*,*θ*,*a*,*b* and *c*.

## 5 Statistical properties

This section is devoted to studying statistical properties of the (*McEG*) distribution, specifically quantile function, moments and moment generating function

### 5.1 Quantile function and simulation

The quantile function corresponding to (7) is *F*(*x*_*q*_) = *P*(*X* ≤ *x*_*q*_) where (*x*_*q*_)_(*McEG*)_ = *F* ^-1^(*u*),is given by the following relation
16


Simulating the *McEG* random variable is straightforward. Let *U* be a uniform variate on the unit interval (0, 1). Thus, by means of the inverse transformation method, we consider the random variable *X* given by the relation
17


### 5.2 Moments

In this subsection we discuss the *r*_*th*_ moment for (*McEG*) distribution. Moments are necessary and important in any statistical analysis, especially in applications. It can be used to study the most important features and characteristics of a distribution (e.g., tendency, dispersion, skewness and kurtosis). We use the results presented earlier, which was obtained by expanding the pdf.

**Theorem 3.1.** If *X* has *McEG*(*φ*,*x*),*φ* = (*λ*,*θ*,*a*,*b*,*c*) then the *r*_*th*_ moment of *X* is given by the following
18


where



**Proof.** Let *X* be a random variable with density function (7). The *r*_*th*_ ordinary moment of the (*McEG*) distribution is given by
19


Using the fact that
20


we obtain
21


again using the binomial series expansion
22


but
23


thus Equation 21 becomes



let *λ*(*k* + 1)*x* = *t* then
24


which completes the proof. Based on the first four moments of the (*McEG*) distribution, the measures of skewness *A*(*φ*) and kurtosis *k*(*φ*) of the (*McEG*) distribution can obtained as
25


and
26


## 5.3 Moment generating function

In this subsection we derived the moment generating function of (*McEG*) distribution.

**Theorem 3.2.** If *X* has (*McEG*) distribution, then the moment generating function *M*_*X*_(*t*) has the following form
27


**Proof.** We start with the well known definition of the moment generating function given by
28


let *x*(*λ*(*k* + 1)-*t*) = *z* then



which completes the proof.

## 6 Conditional moments, residual life and reversed failure rate function

For lifetime models, it is also of interest to find the conditional moments and the mean residual lifetime function. The conditional moments for (*McEG*) distribution is given by
29


using (20), (22) and (23), Equation 29 becomes
30


where



Given that a component survives up to time *t* ≥ 0, the residual life is the period beyond t until the time of failure and defined by the conditional random variable *X* - *t*|*X* > *t*. In reliability, it is well known that the mean residual life function and ratio of two consecutive moments of residual life determine the distribution uniquely (Gupta and Gupta, 1983). Therefore, we obtain the *r* ^*th*^-order moment of the residual life via the general formula



Applying the binomial expansion of (*x*-*t*)^*r*^ and substituting *f*(*x*,*φ*) given by (7) into the above formula gives
31


where  is the upper incomplete gamma function. Also the mean residual life of the *McEG* distribution is given by
32


On the other hand, we analogously discuss the reversed residual life and some of its properties. The reversed residual life can be defined as the conditional random variable *t* - *X*|*X* ≤ *t* which denotes the time elapsed from the failure of a component given that its life is less than or equal to t. This random variable may also be called the inactivity time (or time since failure); for more details you may see (Kundu and Nanda, 2010; Nanda, Singh, Misra, and Paul, 2003). Also, in reliability, the mean reversed residual life and ratio of two consecutive moments of reversed residual life characterize the distribution uniquely. the reversed failure (or reversed hazard) rate function is given by Equation 11. The *r* ^*th*^-order moment of the reversed residual life can be obtained by the well known formula
33


Applying the binomial expansion of (*t*-*x*)^*r*^ and substituting *f*(*x*,*φ*) given by (2.1) into the above formula gives
34


where  is the lower incomplete gamma function. Thus the mean of the reversed residual life of the *McEG* distribution is given by



Using *m*(*t*)and *m*_2_(*t*) we obtain the variance of the reversed residual life of the *McEG* distribution, and hence the coefficient of variation of the reversed residual life of the *McEG* distribution can be easily obtained.

## 7 Distribution of the order statistics

In this section, we derive closed form expressions for the pdfs of the *r*_*th*_ order statistic of the (*McEG*) distribution, also, the measures of skewness and kurtosis of the distribution of the *r*_*th*_ order statistic in a sample of size *n* for different choices of *n*;*r* are presented in this section. Let *X*_1_,*X*_2_,…,*X*_*n*_ be a simple random sample from (*McEG*) distribution with pdf and cdf given by (7) and (9), respectively.

Let *X*_1_,*X*_2_,…,*X*_*n*_ denote the order statistics obtained from this sample. We now give the probability density function of *X*_*r*:*n*_, say *f*_*r*:*n*_(*x*,*φ*) and the moments of *X*_*r*:*n*_, *r* = 1,2,…,*n*. Therefore, the measures of skewness and kurtosis of the distribution of the *X*_*r*:*n*_ are presented. The probability density function of *X*_*r*:*n*_ is given by
35


where *F*(*x*,*φ*) and *f*(*x*,*φ*) are the cdf and pdf of the (*McEG*) distribution given by (7), (8), respectively, and since 0<*F*(*x*,*φ*) < 1, for *x* > 0, by using the binomial series expansion of [1 - *F*(*x*,*φ*)]^*n*-*r*^, given by
36


we have
37


substituting from (7) and (8) into (37), we can express the *k*_*th*_ ordinary moment of the *r*_*th*_ order statistics *X*_*r*:*n*_ say  as a liner combination of the *k*_*th*_ moments of the (*McEG*) distribution with different shape parameters. Therefore, the measures of skewness and kurtosis of the distribution of *X*_*r*:*n*_ can be calculated.

## 8 Estimation and inference

In this section, we determine the maximum likelihood estimates (MLEs) of the parameters of the (*McEG*) distribution from complete samples only. Let *X*_1_,*X*_2_,…,*X*_*n*_ be a random sample of size *n* from *McEG* (*λ*,*θ*,*a*,*b*,*c*).The likelihood function for the vector of parameters *φ* = (*λ*,*θ*,*a*,*b*,*c*) can be written as
38


Taking the log-likelihood function for the vector of parameters *φ* = (*λ*,*θ*,*a*,*b*,*c*) we get
39


The log-likelihood can be maximized either directly or by solving the nonlinear likelihood equations obtained by differentiating (39). The components of the score vector are given by
40
41
42
43


and
44


We can find the estimates of the unknown parameters by maximum likelihood method by setting these above non-linear Eqs. 40- (44) to zero and solve them simultaneously. Therefore, we have to use mathematical package to get the MLE of the unknown parameters. Also, all the second order derivatives exist. Thus we have the inverse dispersion matrix is given by
45



The elements of Hessian matrix is given in the Appendix.

By solving this inverse dispersion matrix these solutions will yield asymptotic variance and covariances of these ML estimators for ,, ,  and  Using (44), we approximate 100(1 - *γ*)*%* confidence intervals for *λ*,*θ*,*a*,*b* and *c* are determined respectively as



where *z*_*γ*_ is the upper 100*γ*_*the*_ percentile of the standard normal distribution.

We can compute the maximized unrestricted and restricted log-likelihood functions to construct the likelihood ratio (LR) test statistic for testing on some the McEG sub-models. For example, we can use the LR test statistic to check whether the McEG distribution for a given data set is statistically *superior* to the EG distribution. In any case, hypothesis tests of the type *H*_0_:*φ* = *φ*_0_ versus *H*_0_:*φ* ≠ *φ*_0_ can be performed using a LR test. In this case, the LR test statistic for testing *H*_0_ versus *H*_1_ is , where  and  are the MLEs under *H*_1_ and *H*_0_, respectively. The statistic *ω* is asymptotically (as *n* → *∞*) distributed as , where *k* is the length of the parameter vector *θ* of interest. The LR test rejects *H*_0_ if , where  denotes the upper 100*γ* *%* quantile of the  distribution.

## 9 Application

In this section, we compare the results of fitting the McEG and EG distributions to real data sets. Sixty-three breaking strengths of glass fibres of length 1.5 cm were reported by Smith and Naylor (1987). No units for the breaking strengths were given. The The data are as follows:



The LR test statistic to test the hypotheses *H*_0_:*a* = *b* = *c* = 1 versus *H*_1_:*a* ≠ 1 ∨ *b* ≠ 1 ∨ *c* ≠ 1 is , so we reject the null hypothesis.

In order to compare the two distribution models, we consider criteria like -2*ℓ*, AIC (Akaike information criterion)and CAIC (corrected Akaike information criterion) for the data set. The better distribution corresponds to smaller -2*ℓ*, AIC and CAIC values:



where *k* is the number of parameters in the statistical model, *n* the sample size and *ℓ* is the maximized value of the log-likelihood function under the considered model. Also, here for calculating the values of KS we use the sample estimates of *θ*,*α*,*a*,*b* and *c*. Table 1 shows the MLEs under both distributions, Table 2 shows the values of -2*ℓ*, AIC and CAIC values. The values in Table 2 indicate that the McEG distribution leads to a better fit than the EG distribution.

**Figure 5:**
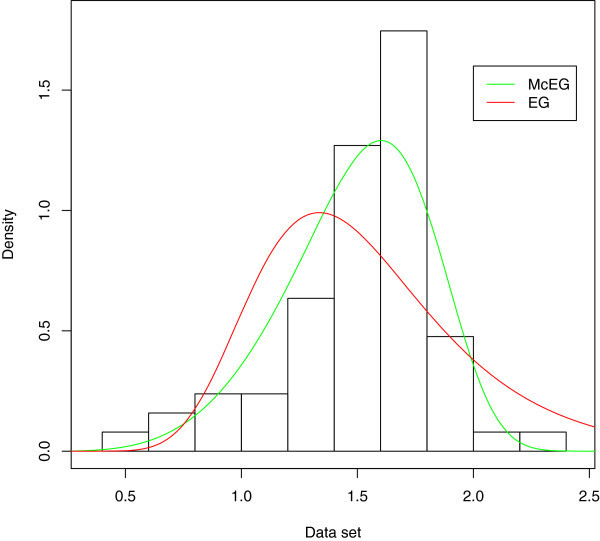
**Estimated densities of the data set.**

A density plot compares the fitted densities of the models with the empirical histogram of the observed data (Figure 5). The fitted density for the McEG model is closer to the empirical histogram than the fits of the EG model.

**Figure 6:**
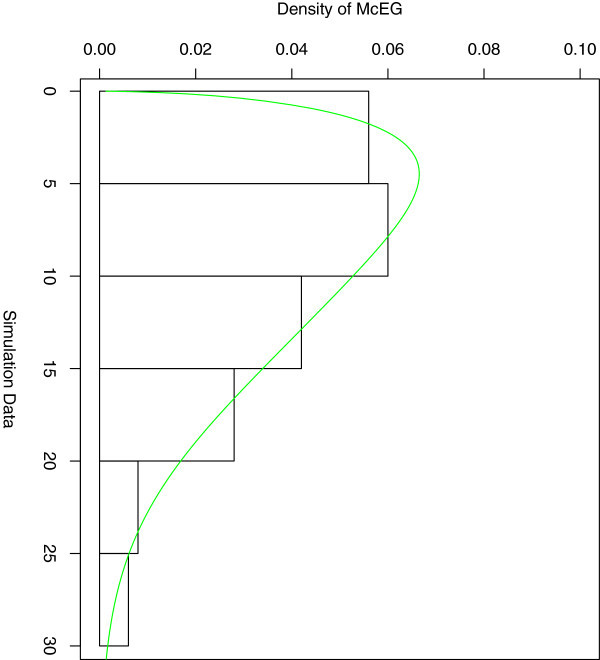
**Empirical, fitted McEG and EG cdf of the data set.**

**Figure 7:**
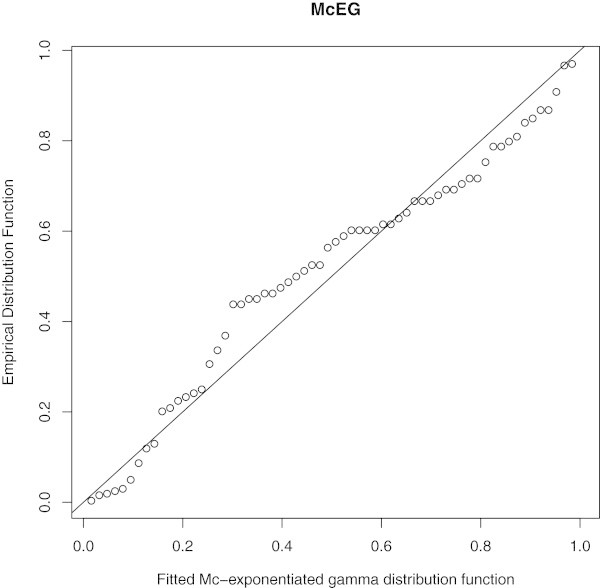
**PP of McEG distribution.**

**Figure 8:**
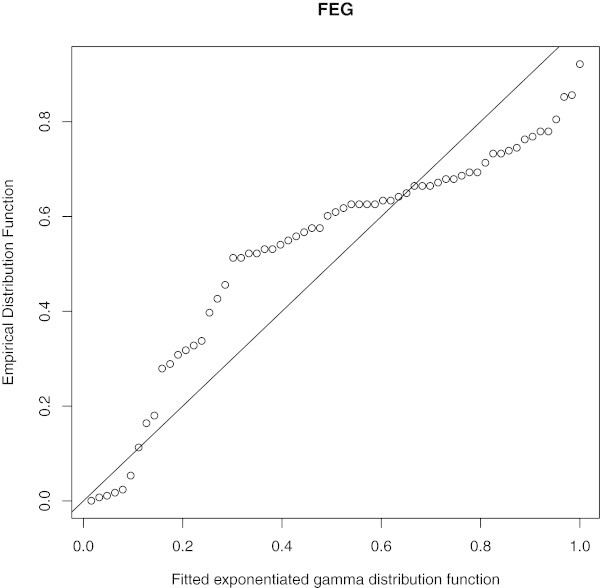
**PP of EG distributions.**

**Figure 9:**
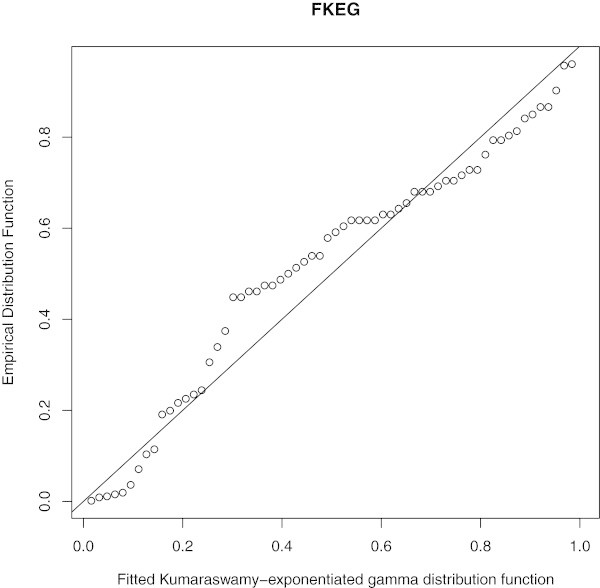
**PP of KEG distributions.**

Empirical, fitted McEG and EG cdf of the data set is given in Figure 6. PP of McEG, EG and KEG distribution are given, respectively in Figures 6, 7, 8 and 9.

**Table 1:** **Estimated parameters of the EG and McEG distribution for the data set**

Model	Parameter Estimate(St. Err)	***-ℓ(·;x)***
EG		30.080
KEG		15.962
		
McEG		14.852

**Table 2:** **Criteria for comparison**

Model	KS	-2 ***ℓ***	AIC	CAIC
EG	0.211	60.161	64.161	64.361
KEG	0.146	31.925	39.925	40.615
McEG	0.139	29.704	39.704	40.578

## 10 Simulated data

In this subsection, we provided an algorithm to generated a random sample from the McEG distribution for the given values of its parameters and sample size *n*. The simulation process consists the following steps:

1. Set *n*, and Θ = (*λ*,*θ*,*a*,*b*,*c*).
2. Set initial value *x* ^0^ for the random starting.
3. Set *j* = 1.
4. Generate *U* ∼ *Uniform* (0,1).
5. Update *x* ^0^ by using the Newton’s formula such as

6. If ∣*x* ^0^-*x* ^⋆^∣ ≤ *ε*, (very small, *ε* > 0 tolerance limit). Then, *x* ^⋆^ will be the desired sample from *F*(*x*).
7. If ∣*x* ^0^-*x* ^⋆^∣ > *ε*, then, set *x* ^0^ = *x* ^⋆^ and go to step 5.
8. Repeat steps 4-7, for *j* = 1,2,…,*n* and obtained *x*_1_,*x*_2_,…,*x*_*n*_.

Using the above algorithm, we generated a sample of size 100 from McEG distribution for arbitrary values of *λ* = 0.1,*θ* = 0.5,*a* = 0.3,*b* = 4 and *c* = 5. The simulated sample is given by



The maximum likelihood estimates with corresponding confidence intervals are calculated based on the simulated sample. The MLEs of (*λ*,*θ*,*a*,*b*,*c*) are



respectively. The asymptotic confidence intervals for (*λ*,*θ*,*a*,*b*,*c*) are obtained as (0 ∼ 0.278), (0 ∼ 13.295), (0 ∼ 0.444), (0 ∼ 8.342) and (0 ∼ 21.30274044) respectively.

**Figure 10:**
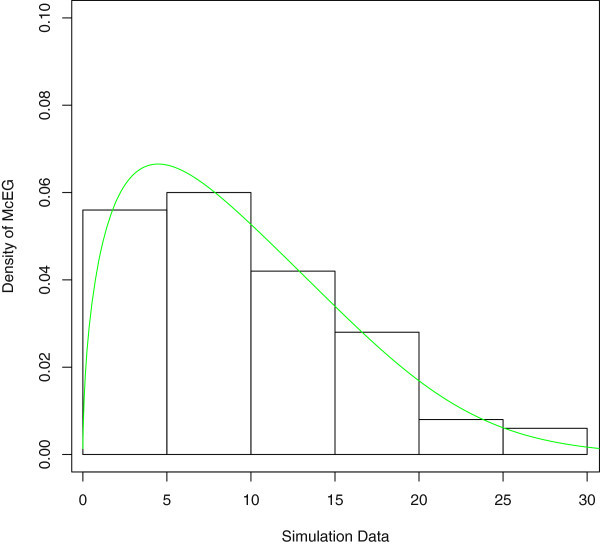
**Estimated pdf of the simulated data.**

**Figure 11:**
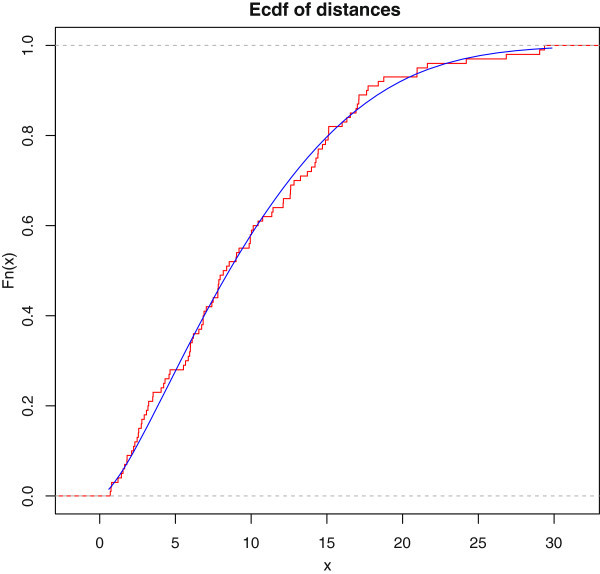
**Empirical, fitted McEG cdf of the simulated data.**

The pdf and empirical, fitted McEG cdf of the simulated data are given in (Figure 10) and (Figure 11).

## 11 Conclusion

Here we propose a new model, the so-called the McEG distribution which extends the EG distribution in the analysis of data with real support. An obvious reason for generalizing a standard distribution is because the generalized form provides larger flexibility in modeling real data. We derive expansions for the moments and for the moment generating function. The estimation of parameters is approached by the method of maximum likelihood, also the information matrix is derived. We consider the likelihood ratio statistic to compare the model with its baseline model. An application of the McEG distribution to real data show that the new distribution can be used quite effectively to provide better fits than EG distribution.

## Appendix

The elements of Hessian matrix are:



